# Renal trauma in a pediatric Level 1 trauma center: morbidity, mortality, and the role of renal scintigraphy

**DOI:** 10.1007/s00068-026-03341-y

**Published:** 2026-10-09

**Authors:** Sem A. M. Jansen, Bouchra Haouli, Manouk Backes, Tjarda N. Tromp, Dennis Vriens, Michael J. R. Edwards, Allon van Uitert, Stijn D. Nelen

**Affiliations:** 1 Department of Trauma Surgery, Radboud University Medical Center, Nijmegen, The Netherlands; 2 Department of Pediatric Surgery, Radboud University Medical Center, Nijmegen, The Netherlands; 3 Department of Medical Imaging, Section of Nuclear Medicine, Radboud University Medical Center, Nijmegen, The Netherlands; 4 Department of Pediatric Urology, Radboud University Medical Center /Amalia Children’s Hospital, Nijmegen, The Netherlands

**Keywords:** Kidney injuries, Pediatric trauma, Renal scintigraphy, Follow-up studies, Hypertension

## Abstract

**Purpose:**

Pediatric renal trauma is uncommon but potentially serious, and is often managed conservatively. Complications may occur, and the clinical value of renal scintigraphy during follow-up remains unclear. This study aimed to assess morbidity and mortality after pediatric renal trauma and to assess the clinical value of renal scintigraphy during follow-up.

**Methods:**

This retrospective single-center cohort study included pediatric trauma patients (< 18 years) with renal injuries admitted to a Level 1 trauma center in the Netherlands between January 1, 2001, and December 31, 2024. Mortality and in-hospital complications were assessed, and associations between renal scintigraphy abnormalities and complications during follow-up were evaluated.

**Results:**

A total of 72 patients were included, of whom all injuries resulted from blunt trauma. Conservative management was applied in 88% of cases; embolization and nephrectomy were performed in 8% and 3% of patients, respectively. In-hospital mortality was 11% and was attributed exclusively to concomitant traumatic brain injury. Complications occurred in 25% of patients, primarily infections (19%), followed by hypertension during follow up (4%). Renal scintigraphy was performed in 53% of patients, predominantly in those with high-grade injuries; abnormalities were frequently observed (77%) but did not correlate with the occurrence of hypertension, proteinuria, or renal insufficiency during follow-up.

**Conclusion:**

Pediatric renal trauma, including high-grade injuries, can often be managed conservatively, and most complications can be treated using medical therapy or minimally invasive interventions. Renal scintigraphy showed no clear association with long-term morbidity and had limited additional value in routine follow-up. Given the potential for late-onset hypertension, structured long-term clinical surveillance with periodic monitoring of blood pressure, proteinuria, and renal function is warranted, particularly after high-grade injuries.

**Supplementary Information:**

The online version contains supplementary material available at https://doi.org/10.1007/s00068-026-03341-y.

## Background

Renal trauma in children is relatively rare, with a reported incidence ranging from 1.2% up to 2.7% of all pediatric trauma admissions, depending on the study population and injury mechanism. Mortality rates have been reported between 3.3% and 10.4%, often attributed to concomitant injuries rather than the renal injury itself [1–3]. Traumatic renal injury is most commonly caused by blunt trauma and is frequently associated with concomitant intra-abdominal injuries, particularly involving the liver and spleen [2, 4, 5].

Pediatric kidneys are relatively large and often retain foetal lobulations, an immature morphological feature that increases their vulnerability to parenchymal injury. Moreover, they are surrounded by less perinephric fat. In addition, the abdominal wall is thinner and less muscular, and the thoracic cage more compliant, making the upper abdomen more vulnerable to injury. These features collectively increase the risk of renal injury during blunt abdominal trauma compared to adults [2, 6, 7]. Most pediatric renal injuries can be managed conservatively, as this approach has proven to be safe and effective, even for higher-grade injuries [2, 3]. Regardless of the management approach, complications such as urinary fistula, urinoma, calculus formation, hydronephrosis, sepsis, chronic pyelonephritis, pseudoaneurysm, and hypertension may occur [1, 2].

Traumatic injury of the kidney may lead to impairment or failure of the affected kidney. Renal scintigraphy, such as ^99m^Tc-radiolabelled dynamic mercaptoacetyltriglycine (MAG3) renography and dimercaptosuccinic acid (DMSA) scanning, can be used during follow-up to assess renal perfusion, function, and cortical uptake, providing insight into potential kidney damage [8–10]. While MAG3 scintigraphy is able to assess perfusion, extraction and excretion including leakage, DMSA scintigraphy focusses merely on functional cortex and is able to detect cortical scarring with higher accuracy [11].

To date, the literature on the predictive value of post-traumatic renal scintigraphy for predicting long-term morbidity in children is limited. Existing studies primarily focus on hypertension and urinary leakage [12–15]. Therefore, the aim of this study was to investigate the incidence of morbidity and mortality following pediatric renal trauma and to evaluate the clinical value of renal scintigraphy during follow-up in identifying patients at risk of developing long-term post-traumatic complications.

## Methods

### Study design

A retrospective single-center cohort study was conducted at a Level 1 trauma center The Netherlands. Pediatric trauma patients under 18 years of age who sustained renal trauma and were admitted to the Emergency Department between January 1, 2001, and December 31, 2024, were included. Patients were identified through the institutional trauma registry, which is used for local trauma registration and contributes data to the Dutch National Trauma Registry (DNTR). Additional data were extracted from electronic patient records. Patients who had registered an objection to the use of their data for scientific research were excluded.

### Data collection

Patient characteristics obtained from the institutional trauma registry included age, sex, and the American Society of Anaesthesiologists (ASA) score. Injury-related variables were the Abbreviated Injury Scale (AIS), Injury Severity Score (ISS), mechanism of injury (blunt or penetrating), and cause of injury (e.g., traffic accident, blunt impact, low- or high-energy fall, or other). Additional data extracted from the electronic medical records included shock status on emergency department arrival (defined as the need for blood transfusion and an elevated pediatric-adjusted shock index), the American Association for the Surgery of Trauma Organ Injury Scale (AAST-OIS) grade for renal trauma, side of the injured kidney, and the presence of concomitant intra-abdominal injuries [16, 17]. Renal trauma management strategies (conservative management, ureteral stenting, nephrostomy, nephrectomy, embolization/coiling, or abdominal packing) and renal scintigraphy findings during follow up, were also recorded. Included outcomes were complications, in-hospital mortality, intensive care unit (ICU) length of stay and total hospital length of stay (LOS). Long-term follow-up was primarily obtained through telephone interviews assessing current health status and antihypertensive medication use. When patients remained under active outpatient follow-up, data were obtained from the electronic medical record. For patients who could not be contacted and were no longer under hospital follow-up, the last documented outpatient visit was considered the end of follow-up.

### Primary and secondary outcomes

Primary outcomes were in-hospital mortality and complications during hospital admission following renal trauma. The secondary outcome was the association between abnormalities on renal scintigraphy and the occurrence of complications during follow up, including hypertension, proteinuria and renal insufficiency.

Hypertension was defined as elevated blood pressure requiring antihypertensive treatment.

Renal insufficiency was classified based on the estimated glomerular filtration rate (eGFR): mild (60–89 mL/min/1.73 m²), moderate (30–59 mL/min/1.73 m²), severe (15–29 mL/min/1.73 m²), and renal failure (< 15 mL/min/1.73 m² or requiring dialysis) [18].

Other complications included infection, urinoma, rebleeding, perinephric abscess, urinary fistula, arteriovenous fistula, and hydronephrosis. These were only considered complications if they were diagnosed, treated, and explicitly documented as such in the electronic medical record by a healthcare professional.

### Renal scintigraphy

In this Level 1 trauma center, there was no strict follow-up protocol during the study period. In general, patients with moderate- to high-grade renal injuries were followed by a pediatric urologist after discharge. At the discretion of the treating pediatric urologist, renal and bladder ultrasonography was commonly performed approximately 6–12 weeks after injury to assess anatomical recovery, and renal scintigraphy (MAG3 or DMSA) could be performed thereafter to evaluate differential renal function and cortical scarring, as both may represent risk factors for subsequent hypertension, proteinuria, and impaired renal function. For the purpose of this study, all available renal scintigraphy images were reassessed by a trauma surgeon and pediatric urologist. Split renal function was classified as moderate impairment (30–<40% split function), severe impairment (10–<30%), or afunctional kidney (< 10%). Renal scarring was defined as diminished or absent focal cortical tracer uptake. Impaired excretion was interpreted as obstruction or injury of the renal pelvis or ureter and was assessable only with MAG3 scintigraphy. Based on the reassessment, patients were classified as having either normal or abnormal renal scintigraphy results.

### Statistical analysis

Descriptive statistics were used to characterize the study population and the primary and secondary outcomes. Categorical variables were reported as frequencies and percentages, and continuous variables as means with standard deviations or medians with interquartile ranges (25th to 75th percentile), depending on the distribution. Normality was assessed using the Shapiro–Wilk test. Associations between renal scintigraphy abnormalities and the occurrence of complications were evaluated using Fisher’s exact test. A two-sided p-value of less than 0.05 was considered statistically significant. All analyses were performed using SPSS Statistics, (version 31.0; IBM SPSS Statistics, Armonk, NY).

### Ethics approval

The local Medical Ethics Committee confirmed that the study was not subject to the Medical Research Involving Human Subjects Act (WMO), and therefore formal ethical approval was not required. Informed consent was obtained from patients and/or their legal guardians when applicable.

## Results

A total of 85 pediatric patients met the inclusion criteria. Thirteen patients were excluded due to lack of informed consent or incomplete documentation, resulting in a final cohort of 72 patients. The majority were male (54%), with a median age of 14 years (IQR 9–16). All injuries resulted from blunt trauma, most commonly caused by traffic accidents (60%). Most often, the abdomen was the most severely affected body region (78%), followed by the thorax (44%) and head (26%). A total of 83% of patients sustained severe injuries, defined as an ISS of 16 or higher. Nine patients (13%) presented in shock upon arrival at the emergency department, of whom three died during admission. All three deaths were attributable to severe traumatic brain injury, with an immediately infaust neurological prognosis at presentation (Table 1).

**Table 1:** Baseline characteristics of pediatric patients with renal trauma (*N* = 72)

Characteristic		Median (IQR)/ Numbers (%)
Age at time of injury (years)		14 (9–16)
Gender	Male	39 (54)
	Female	33 (46)
ASA physical status score	I Normal health	65 (90)
	II Mild systemic disease	3 (4)
	III Severe systemic disease	4 (6)
Mechanism of injury	Blunt	72 (100)
	Penetrating	0 (0)
Injury cause	Traffic accident	43 (60)
	High-energy fall	11 (15)
	Low-energy fall	7 (10)
	Blunt object-related injury	6 (8)
	Other	5 (7)
AIS ≥ 3 per body region	Head	19 (26)
	Face	1 (1.4)
	Neck	2 (3)
	Thorax	32 (44)
	Abdomen	56 (78)
	Spine	2 (3)
	Upper extremity	2 (3)
	Lower extremity	14 (19)
ISS	ISS ≤ 8	4 (6)
	ISS 9–15	8 (11)
	ISS 16–24	30 (42)
	ISS ≥ 25	30 (42)
Shock on arrival at ED	Yes	9 (13)
	No	62 (86)
Renal scintigraphy performed (among survivors, *n* = 64)	MAG3	29 (45)
	DMSA	5 (8)

Values are presented as median (IQR) or numbers (%)

ASA = American Society of Anesthesiologists; ISS = Injury Severity Score; ED = Emergency Department; MAG3 = mercaptoacetyltriglycine; DMSA = dimercaptosuccinic acid

Renal trauma was non-isolated in most cases, with 68% of patients sustaining concomitant intra-abdominal injuries. The spleen (44%) and liver (30%) were the most frequently affected organs. Hematuria occurred in 53% of patients, with 26% requiring bladder catheterization. In total, 29% of patients received a blood transfusion during admission. The majority of patients (88%) were treated conservatively. Nephrectomy was performed in two patients (3%), and embolization was required in six (8%). The median hospital length of stay was 8 days (IQR 2–15), and the median ICU stay was 1 day (IQR 1–2) (Table 2).

**Table 2:** Injury and management characteristics of pediatric patients with renal trauma (*N* = 72)

Characteristic		Median (IQR)/ Numbers (%)
LOS on ICU (days)		1 (1–2)
LOS in hospital (days)		7 (2–15)
Duration of bed rest (days)		2 (1–8)
Duration of restriction from contact sports (weeks)		6 (4–6)
Hematuria	Yes	38 (53)
	Unknown	13 (18)
	Macroscopic	30 (42)
	Microscopic	7 (10)
	Catheterization required	19 (26)
AAST Grade kidney injury	I	10 (14)
	II	15 (21)
	III	17 (24)
	IV	24 (33)
	V	6 (8)
Affected kidney	Left	43 (60)
	Right	24 (33)
	Bilateral	5 (7)
Management of renal trauma	Conservative management	63 (88)
	Coiling/embolization	6 (8)
	Thrombectomy	1 (1)
	Renal artery anastomosis	1 (1)
	Ureteral stent (JJ-Stent)	1 (1)
	Abdominal packing	1 (1)
	Nephrectomy	2 (3)
Blood transfusion during admission		21 (29)
Concomitant intra-abdominal injury	Isolated renal injury	23 (32)
	Single concomitant abdominal injury	34 (47)
	Multiple (≥ 2 concomitant abdominal injuries)	15 (21)
	*Injured organs*	
	Spleen	32 (44)
	Liver	21 (30)
	Pancreas	3 (4)
	Small bowel	3 (4)
	Vena cava	2 (3)
	Adrenal glands	1 (1)
	Ureter	1 (1)
	Bladder	1 (1)

Values are presented as median (IQR) or numbers (%)

LOS = length of stay; ICU = intensive care unit; AAST = American Association for the Surgery of Trauma

Complications were observed in 18 patients (25%), of whom 83% developed complications during hospital admission and 17% during follow-up; 6% experienced complications during both periods. The predominant complications were infections (19%), of which urinary tract infections that required antibiotic treatment were most frequent. One patient developed a retroperitoneal abscess following nephrectomy, which required surgical drainage. Another patient developed an infected urinoma following initial embolization, which was treated with percutaneous drainage and ureteral stenting. Additionally, one patient underwent nephrectomy nine days after embolization due to necrosis of the embolized kidney.

The overall in-hospital mortality rate was 11% (*n* = 8). All deceased patients had sustained very severe injuries (ISS ≥ 25) and had concomitant head trauma with an AIS ≥ 3. None of the deaths were primarily attributed to renal trauma. Among the deceased patients, only one patient had a high-grade renal injury (AAST grade IV–V) (Table 3).

**Table 3:** Clinical outcomes of pediatric patients with renal trauma (*N* = 72)

Clinical outcome		Median (IQR)/ Numbers (%)
In-hospital mortality		8 (11)
Follow-up (months) (*N* = 64)		42.5 (8.5–111.0)
Complications	No	54 (75)
	Yes	18 (25)
	Infection	14 (19)
	Urinary tract infection	6 (8)
	Infected urinoma	1 (1)
	Infection of unknown primary	5 (7)
	Pulmonary infection	2 (3)
	Abscess	1 (1)
	Hypertension	3 (4)
	Decreased kidney function	1 (1)
	Proteinuria	3 (4)

Values are presented as median (IQR) or numbers (%)

In patients with available follow-up data, hypertension during follow-up occurred in three patients (4%), all of whom had sustained high-grade (AAST grade IV–V) renal injuries and required antihypertensive medication. One patient developed hypertension and concominant proteinuria one year after conservatively treated grade V renal trauma at the age of five. A second patient developed hypertension at the age of 13, six years after conservative management of a grade IV renal injury; proteinuria was also observed during follow-up. The third patient developed hypertension at the age of 28, eleven years after nephrectomy for a grade V injury, and had proteinuria and mild renal insufficiency during follow-up.

### Renal scintigraphy

Renal scintigraphy was performed in 34 patients of the 64 surviving patients (53%) during outpatient follow-up, approximately 6 to 12 weeks post-injury, including five DMSA scans and 29 MAG3 scans. Of these 34 patients, 25 (74%) had sustained high-grade renal injuries (AAST IV–V). Five patients with high-grade injuries (13%) did not undergo renal scintigraphy. Abnormal findings were observed in 26 scans (77%), the majority of which (73%) occurred in patients with high-grade injuries.

Notably, in five patients with high-grade trauma, no abnormalities were detected. Impaired ipsilateral renal function was observed in 35% of all scans, reduced cortical uptake in 74%, and delayed excretion in one case (Table 4).

**Table 4:** Renal scintigraphy abnormalities in pediatric patients with renal trauma (*N* = 34)

Abnormalities		Numbers (%)
Split function	No impairment	22 (65)
	Moderately impaired	3 (9)
	Severely impaired	5 (15)
	Afunctional	3 (9)
	Unmeasurable*	1 (3)
Decreased/absent focal cortical uptake	Yes	26 (77)
	No	8 (24)
	Unknown	1 (3)
Excretion	Delayed (MAG3)	4 (12)
	Normal (MAG 3)	25 (74)
	Not applicable (DMSA)	5 (15)

MAG3 = mercaptoacetyltriglycine; DMSA = dimercaptosuccinic acid

* Split renal function was considered unreliable in one patient because a pelvic kidney was partially obscured by the overlying spine, precluding accurate differential renal function assessment

There were no significant differences in patient characteristics or outcomes, between patients with abnormal and normal scintigraphy findings (see Supplemental Digital Content 1, Table, which summarizes patient characteristics and outcomes stratified by scintigraphy findings). Overall complication rates were similar between patients with abnormal and normal scintigraphy findings (46% vs. 43%, *P* = 1.000; Table 5). However, none of the patients with normal renal scintigraphy developed hypertension, proteinuria, or renal insufficiency during follow-up. Although these complications occurred exclusively in patients with abnormal scintigraphy findings, the association did not reach statistical significance (Table 5).

**Table 5:** a. Association between renal scintigraphy abnormalities and complications in pediatric patients with renal trauma (*N* = 33). b. Association between renal scintigraphy abnormalities and hypertension, renal insufficiency and proteinuria in pediatric patients with renal trauma (*N* = 33)

	Complications	No complications	P value
	Numbers (%)		
**a**			
Abnormal renal scintigraphy	12 (46)	14 (54)	1.000
Normal renal scintigraphy	3 (43)	4 (57)	
**b**			
Abnormal renal scintigraphy	2 (8)	24 (92)	1.000
Normal renal scintigraphy	0 (0)	7 (100)	

Of the 34 renal scintigraphy scans performed, five did not lead to any further clinical recommendations. In the remaining 29 patients, a recommendation for routine or annual blood pressure monitoring was documented, irrespective of whether scintigraphy findings were normal or abnormal. The advised monitoring interval and setting varied and included home measurements, follow-up by the general practitioner, or evaluation by a pediatrician.

## Discussion

This single-center cohort study demonstrated that conservative management of renal injury in pediatric patients is safe and effective, even for high-grade cases. The high in-hospital mortality rate reflects the overall severity of trauma and the impact of concomitant injuries; however, no deaths were attributed to renal trauma itself. Abnormal findings on renal scintigraphy during follow-up were not associated with the occurrence of hypertension, impaired renal function, or proteinuria. Within the limitations of this retrospective study, no apparent differences in subsequent clinical management were identified between patients with normal and abnormal scintigraphy findings.

Consistent with current literature, conservative management has become the standard of care for hemodynamically stable children with renal trauma [19–21]. In our cohort most patients were managed conservatively, with low rates of nephrectomy and embolization. Despite this predominantly conservative approach, blood transfusions were frequently required, likely reflecting the overall trauma burden rather than kidney-specific injury. Renal trauma was non-isolated in the majority of cases and was frequently accompanied by concomitant intra-abdominal injuries, most commonly involving the spleen and liver, which are commonly affected in pediatric blunt abdominal trauma [22].

The relatively high in-hospital mortality in this cohort likely reflects the severity of injury and the substantial burden of concomitant injuries in this predominantly level 1 trauma center population [1, 9, 23, 24]. Importantly, all deaths were attributable to severe traumatic brain injury, which remains a leading cause of mortality in pediatric trauma [25, 26]. Hospital and ICU length of stay were comparable to those reported in pediatric renal trauma cohorts, suggesting that conservative management did not result in prolonged hospitalization [1, 3, 23].

The most common complications were infection-related and were generally managed conservatively with antibiotics or, in some cases, minimally invasive interventions such as percutaneous drainage. Hypertension occurred exclusively in patients with high-grade injuries and was observed at a rate consistent with the lower end of those reported in prior studies (0–15%) [3, 13, 27–30]. Notably, in this cohort, hypertension was often diagnosed several years after the initial renal injury, whereas previous literature suggests that patients who are normotensive in the immediate post-trauma period are unlikely to develop delayed-onset hypertension during follow-up [7, 13].

Although visible hematuria is more commonly associated with high-grade renal injuries than microscopic hematuria, it is not sufficiently reliable to accurately determine injury severity. In this cohort, visible hematuria was present in only three-quarters of patients with high-grade injuries, indicating that the absence of visible hematuria does not exclude significant renal damage. These findings are consistent with previous literature and underscore the importance of considering clinical factors such as injury mechanism, hemodynamic status, and physical findings when evaluating pediatric abdominal trauma, rather than relying solely on hematuria to guide decisions regarding further imaging, including computed tomography [3, 7, 9].

Renal scintigraphy was predominantly performed in patients with high-grade injuries; however, scintigraphic abnormalities did not demonstrate a clear association with injury grade or complications. However, hypertension, proteinuria, and reduced renal function were observed exclusively in patients with abnormal renal scintigraphy, whereas in hospital complications occurred in both patients with normal and abnormal scintigraphy findings. These differences did not reach statistical significance, possibly due to the limited sample size. Similar findings were reported by Wong et al., who observed a high prevalence of scintigraphic abnormalities without a clear correlation with clinical outcomes, underscoring the ongoing uncertainty regarding the routine use of renal scintigraphy during follow-up [9]. In line with these findings, scintigraphy results did not appear to influence subsequent follow-up recommendations in this cohort, as routine blood pressure monitoring was advised irrespective of scan abnormalities.

This study was conducted at a single Level 1 trauma center with a relatively small sample size, which may limit the generalizability of the findings. Follow-up was restricted to patients who could be contacted by telephone or had documented follow-up in the electronic patient record, introducing potential selection bias. Renal scintigraphy was not performed routinely but mainly in patients with high-grade injuries, which may have introduced additional selection bias and overestimated the prevalence of abnormal findings. Furthermore, complications such as hypertension and renal insufficiency were captured only in patients with available follow-up and formal physician diagnoses, potentially leading to underestimation of these outcomes.

In conclusion, pediatric renal trauma, including high-grade injuries, can often be managed safely and effectively with a predominantly conservative approach. Renal scintigraphy showed no clear association with long-term morbidity and appears to have limited additional value in routine follow-up. Structured long-term follow-up with periodic monitoring of blood pressure, proteinuria, and renal function may therefore represent a pragmatic follow-up strategy, particularly in patients with high-grade injuries.

## Supplementary Information

Below is the link to the electronic supplementary material.

Supplementary Material 1

## Data Availability

Data are not publicly available due to privacy regulations but may be available from the corresponding author upon reasonable request.
