# Supplementary material for: Renal trauma in a pediatric Level 1 trauma center: morbidity, mortality, and the role of renal scintigraphy
Source: Eur J Trauma Emerg Surg. 2026 Oct 9;52(1):313. doi: 10.1007/s00068-026-03341-y (PMC13652885; doi:10.1007/s00068-026-03341-y)
Supplement: Supplementary file 1 [file 68_2026_3341_MOESM1_ESM.docx]

*Supplementary Information*

European Journal of Trauma and Emergency Surgery

Renal Trauma in a Pediatric Level I Trauma Center: Morbidity, Mortality, and the Role of Renal Scintigraphy

Sem A.M. Jansen et al.

Corresponding author: sem.jansen@radboudumc.nl

**Table 1.** Patient characteristics and outcomes stratified by renal scintigraphy findings (N = 33).

| **Characteristic** | | **Abnormal findings (*N* = 26)** | **Normal findings (*N* = 7)** | **P value** |
| --- | --- | --- | --- | --- |
| Median (IQR)/ Numbers (%) | | | | |
| **Age** | | 10 (8-15) | 8 (7-13) | 0.476 |
| **Gender** | Male | 11 (42) | 2 (29) | 0.676 |
|  | Female | 15 (58) | 5 (71) |  |
| **Shock on arrival at ED** | | 2 (8) | 1 (14) | 0.523 |
| **ISS** | | 16 (16–22) | 21 (16-29) | 0.268 |
| **AAST injury grade** | Low grade (I-III) | 7 (27) | 2 (29) | 1.000 |
|  | High grade (IV-V) | 19 (73) | 5 (71) | 1.000 |
| **Management** | Conservative | 21 (80) | 6 (86) | 1.000 |
|  | Coiling/embolization | 3 (12) | 1 (14) | 1.000 |
| **Blood transfusion during admission** | | 3 (12) | 2 (29) | 0.282 |
| **LOS on ICU (days)** | | 1 (0-2) | 1 (1-4) | 0.308 |
| **LOS hospital (days)** | | 15 (11 – 20) | 14 (6-23) | 1.000 |
| Continuous variables are presented as median (IQR); categorical variables are presented as numbers (percentages).  ED = emergency department; ISS = Injury Severity Score; AAST = American Association for the Surgery of Trauma; LOS = length of stay; ICU = intensive care unit. | | | | |
